# Glanzmann's Thrombasthenia: How Listening to the Patient Is Sometimes the Simple Key to Good Medicine!

**DOI:** 10.1155/2020/4862987

**Published:** 2020-07-10

**Authors:** Thejus T. Jayakrishnan, Vladimir Limonnik, Deep Shah, Prerna Mewawalla

**Affiliations:** Division of Hematology and Cellular Therapy, Department of Medicine, Allegheny General Hospital, Pittsburgh, PA, USA

## Abstract

*Introduction*. Glanzmann's thrombasthenia is a rare clotting disorder caused by impaired platelet function. Lack of awareness of the appropriate management of rare medical conditions may lead to patient dissatisfaction and potentially poor treatment outcome. *Case Report*. A 78-year-old male with a history of Glanzmann's thrombasthenia was admitted to the trauma service following a fall in which he sustained a facial laceration as well as maxillary sinus and nasal fractures. He received DDAVP 20 mcg and tranexamic acid upon presentation to the emergency department (ED). In the ED, the patient requested administration of platelet transfusion but was refused due to a normal platelet count. During the course of his hospital stay, he complained of epistaxis and was noted to have a downtrending hemoglobin from 11.0 g/dl to 9.0 g/dl. The patient and his family were not comfortable when the discharge plan was finalized and demanded platelet transfusion (due to history of needing platelets in association with injuries or procedures in the past) was refused by the primary team as they continued to state that his platelet count is normal. On hospital day 3, hematology was consulted as the patient and his family were extremely angry and hematology recommended platelet transfusion. Further clinical information was not available as the patient was transferred to another facility per family request as they wanted to be at a center which had the patient's primary hematologist. *Discussion*. A delay in specialist consultation resulted in patient dissatisfaction and extended the length of stay. Patients with rare medical conditions and potential for major complications should be managed aggressively with appropriate specialist consultation to promote patient satisfaction and improve the overall quality of care. This case shows that as physicians it our duty to listen to our patient's concerns and involve them in the medical decision-making to provide optimal patient-centered care.

## 1. Introduction

Glanzmann's thrombasthenia (GT) is a rare clotting disorder caused by impaired platelet function [1]. It is caused by a defect and/or deficiency of a platelet integrin, alpha IIB beta 3. This integrin molecule serves as a receptor for fibrinogen and is essential for platelet aggregation in hemostasis [2]. Bleeding involving GT could be variable and sometimes severe [3]. Lack of awareness of the appropriate management may lead to patient dissatisfaction and potentially poor treatment outcome. We hereby outline such a case where delay in expert consultation resulted in a delay in treatment and adverse outcome, namely, patient dissatisfaction.

## 2. Case Report

A 78-year-old male with a history of heart failure status after AICD/PPM, coronary artery disease status after coronary artery bypass graft surgery (×4), percutaneous coronary artery stenting (×5), hypertension, hyperlipidemia, hepatitis C, and importantly Glanzmann's thrombasthenia presented to the hospital after a fall.

The patient reported that he was closing heavy curtains at a rehabilitation facility when he lost his balance causing him to fall and strike the back of his head and right arm. He complained of diffuse pain all over his body especially his face from the fall. His heart rate was 75 beats per minute and blood pressure 150/90 mm Hg, and he had normal oxygen saturation on room air. Examination was positive for left frontal hematoma with significant periorbital swelling, right inner eye laceration with mild oozing, mild epistaxis with dried fresh blood in the posterior oropharynx, hematoma of right posterior forearm, tenderness to palpation of the abdomen, pelvis, and thoracic (T) and lumbar (L) spine with no neurological deficits or peritoneal signs. Laboratory workup showed microcytic anemia with hemoglobin and hematocrit of 11 g/dl and 34% with a mean corpuscular volume of 81 fl and thrombocytopenia with a platelet count of 141 k/mcl. Computed tomography (CT) scan revealed an acute fracture involving the right nasomaxillary suture extending into the frontal process of the right maxillary bone with possible involvement of nasolacrimal duct, possible left nasomaxillary fracture-diastasis, and fluid in the right mastoid air cells with no evidence of temporal bone fracture, sinus fracture, and nasal fractures. CT of the neck, T and L spine, and abdomen pelvis were negative for acute fractures. He was evaluated by an oral maxillofacial surgery team who performed bedside facial laceration repair. He was admitted to the trauma service for monitoring.

In the ED, the patient was concerned that he was swallowing blood and requested administration of platelet transfusion. In addition to the above laboratory workup, he had a thromboelastography (TEG) that showed mild platelet dysfunction consistent with the history of Glanzmann's thrombasthenia reported by the patient. He was given DDAVP 20 mcg and 1000 mg tranexamic acid but refused platelet transfusion. During the course of his hospital stay, he complained of epistaxis and was noted to have downtrending hemoglobin from 11.0 g/dl to 9.0 g/dl. The patient and his family were not comfortable when the discharge plan was finalized and demanded platelet transfusion (due to history of needing platelets in association with injuries or procedures in the past) was refused by the primary team as they continued to state that his platelet count is normal. On hospital day 3, hematology was consulted as the patient and family were extremely upset. Hematology recommended platelet transfusion and the patient was transfused 2 units of platelets. Further clinical information is not available as the patient was transferred to another facility per family request as they wanted to be at a center which had the patient's primary hematologist.

## 3. Discussion

Glanzmann's thrombasthenia (GT) is a congenital clotting disorder first described by Dr. Eduard Glanzmann in 1918 as “hereditary hemorrhagic thrombasthenia” after patients were noted to have a prolonged bleeding time as well as the absence of platelet clumping on peripheral blood smear [4]. The prevalence of GT is estimated to be around one per million individuals in the general population [2]. However, highly consanguineous populations such as Iran, Canada, Newfoundland, and Jordan have an increased prevalence of approximately one per 200,000 individuals [5].

GT is characterized by the inability of platelets to form platelet-to-platelet aggregates and form a platelet plug at the site of blood vessel injury even with the release of physiologic agonists including collagen, thrombin, and adenosine diphosphate. This lack of aggregation is due to an abnormality in the surface adhesion complex glycoprotein (GP) IIb/IIIa receptor, an integrin made up of a heterodimer consisting of alpha IIb and beta_3_ subunits [6]. This integrin functions as a platelet fibrinogen receptor and is crucial for platelet aggregation and promoting hemostasis. Consequently, the lack of platelet aggregation and hemostasis ultimately leads the patients suffering with this disorder to experience bleeding episodes. GT follows an autosomal recessive inheritance pattern and is due to a gene mutation of *ITGA2B* or *ITGB3*. These genes are located on chromosome 17q21 and are responsible for encoding the alpha IIb and beta_3_ subunits. A mutation in either of the two genes may lead to GT; however, the patient must be homozygous for the same mutation or may be compound heterozygous for different mutations from each parent [3]. Although rare, GT may also be acquired and is due to autoantibodies developing against the glycoprotein (GP) IIb/IIIa receptor. This has been documented in individuals who have an underlying hematologic condition such as multiple myeloma and Hodgkin's lymphoma [7].

Bleeding associated with GT ranges from mild to severe. The severity of bleeding and its association to specific GT mutations has not been well established. In one study, individuals with the *α*2 C807 T gene polymorphism exhibited a milder clinical phenotype, but it may not be of much clinical utility in genotyping these patients [8]. According to Glanzmann's Thrombasthenia Registry (GTR) that prospectively studied 218 GT patients, the mean age at which a patient's first bleeding symptoms occurred was around 5.6 years. Bleeding events were typically mucocutaneous and most commonly involved epistaxis (79.2%), gingival bleeding (61.9%), dental bleeding (37.6%), menorrhagia (73.6%), easy bruising/purpura/petechiae (43.1%), subcutaneous hematoma (37.6%), and gastrointestinal bleeding (22.9%) [9]. Patients presenting with these symptoms and in whom one suspects an underlying bleeding disorder should initially be evaluated regarding his or her personal and family history of bleeding and/or bruising. The patient should be questioned about the common manifestations of GT, including epistaxis, gingival bleeding, and menorrhagia [2]. Bleeding assessment tools, such as those developed by the International Society for Thrombosis and Hemostasis, have been validated to objectively obtain a bleeding history [10]. Medications should be reviewed with particular attention to nonsteroidal anti-inflammatory drugs as these therapies are frequent causes for acquired platelet functional defects. Initial laboratory screening tests should include a complete blood count, peripheral blood smear, plasma thromboplastin time (aPTT), thrombin time (PT), fibrinogen level, factor XIII screen, and von Willebrand disease studies, including VWF antigen, ristocetin cofactor activity, and factor VIII activity [5]. If the history and screening tests are in favor of an inherited platelet defect, additional testing such as light transmission aggregometry (LTA) and flow cytometry may be utilized for definitive diagnosis. LTA is considered the gold standard test and will reveal absent response to all agonists except agglutination to ristocetin. Flow cytometry will reveal alpha IIb beta 3 integrin deficiency [11].

The management of GT is based on the severity of the bleeding. Patients with minor bleeding amenable to local control measures may be managed with pressure, ice packs, electrocauterization, and surgical repair [2, 12]. Tranexamic acid which is an antifibrinolytic agent has also been utilized as a local hemostatic agent as in our case [5]. If the patients do not respond appropriately to local hemostatic techniques or if the condition worsens, systemic measures may be pursued. The agents available for systemic management are evolving and include–platelets, antifibrinolytics, and recombinant factor VIIa, either alone or in combination [9]. These agents are also used in prophylaxis during surgical procedures [9].

While the use of platelets have remained standard for both treatment of bleeding and prophylaxis, the use of factor VII either in isolation or in combination with other agents has been shown to be equally efficacious [9, 13]. It is especially effective in cases with platelet refractoriness such as in the presence of platelet antibodies [5, 14]. In addition to refractoriness, the risk of platelet transfusion also includes infectious complications although the incidence may be very low [3]. The mechanism of action of factor VII involves activation of factor X resulting in burst of thrombin generation which in turn converts fibrinogen to fibrin and enhances GT platelet adhesion and aggregation. Thrombin also improves the stability of the final clot structure by different mechanisms [3].

While the common presentations involve minor bleeding episodes, spontaneous or associated with procedures, major life threatening bleeds do occur in these patients [15]. There are no established guidelines for treating these patients, and response to treatment is assessed through a combination of clinical assessment and functional platelet testing [14, 15]. Therefore, care for patients who present with multisystem trauma or require complex management is best coordinated in a multidisciplinary fashion involving expertise of hematologists and transfusion medicine specialists in addition to the primary treatment team [5, 16]. As these patients are used to prolonged bleeding and need for platelet transfusions, lack of timely transfusion in the setting of ongoing bleeding may be anxiety provoking for the patient and the family as in our case.
